# Environmental justice and drinking water quality: are there socioeconomic disparities in nitrate levels in U.S. drinking water?

**DOI:** 10.1186/s12940-018-0442-6

**Published:** 2019-01-17

**Authors:** Laurel A. Schaider, Lucien Swetschinski, Christopher Campbell, Ruthann A. Rudel

**Affiliations:** 1 Silent Spring Institute, 320 Nevada Street, Suite 302, Newton, MA 02460 USA; 2 Environmental Working Group, 1436 U St. NW, Suite 100, Washington, DC 20009 USA

**Keywords:** Agricultural pollution, Community water systems, Disparities, Drinking water, Environmental justice, Exposure, Land use, Nitrate, Safe Drinking Water Act, Water quality

## Abstract

**Background:**

Low-income and minority communities often face disproportionately high pollutant exposures. The lead crisis in Flint, Michigan, has sparked concern about broader socioeconomic disparities in exposures to drinking water contaminants. Nitrate is commonly found in drinking water, especially in agricultural regions, and epidemiological evidence suggests elevated risk of cancer and birth defects at levels below U.S. EPA’s drinking water standard (10 mg/L NO_3_-N). However, there have been no nationwide assessments of socioeconomic disparities in exposures to nitrate or other contaminants in U.S. drinking water. The goals of this study are to identify determinants of nitrate concentrations in U.S. community water systems (CWSs) and to evaluate disparities related to wealth or race/ethnicity.

**Methods:**

We compiled nitrate data from 39,466 U.S. CWSs for 2010–2014. We used EPA’s Safe Drinking Water Information System (SDWIS) to compile CWS characteristics and linked this information with both city- and county-level demographic data gathered from the U.S. Census Bureau. After applying multiple imputation methods to address censored nitrate concentration data, we conducted mixed-effects multivariable regression analyses at national and regional scales.

**Results:**

5.6 million Americans are served by a CWS that had an average nitrate concentration ≥ 5 mg/L NO_3_-N between 2010 and 2014. Extent of agricultural land use and reliance on groundwater sources were significantly associated with nitrate. The percent of Hispanic residents served by each system was significantly associated with nitrate even after accounting for county-level cropland and livestock production, and CWSs in the top quartile of percent Hispanic residents exceeded 5 mg/L nearly three times as often as CWSs serving the lowest quartile. By contrast, the percent of residents living in poverty and percent African American residents were both inversely associated with nitrate.

**Conclusions:**

Epidemiological evidence for health effects associated with drinking water above 5 mg/L NO_3_-N raises concerns about increased risk for the 5.6 million Americans served by public water supplies with average nitrate concentrations above this level. The associations we observed between nitrate concentrations and proportions of Hispanic residents support the need for improved efforts to assist vulnerable communities in addressing contamination and protecting source waters. Future studies can extend our methods to evaluate disparities in exposures to other contaminants and links to health effects.

**Electronic supplementary material:**

The online version of this article (10.1186/s12940-018-0442-6) contains supplementary material, which is available to authorized users.

## Background

Drinking water quality is regulated in the United States under the Safe Drinking Water Act (SDWA), which establishes national monitoring and reporting requirements and maximum contaminant levels (MCLs) for 88 contaminants. In 2013, 9800 public water systems, serving 26.5 million Americans, had violations of health-based standards [1]. In 2015, lead contamination in the drinking water supply of Flint, Michigan, caused elevated blood lead levels in children following the use of a new drinking water source, the Flint River, as a cost-saving measure [2]. Because 60% of Flint’s residents are African American and 40% live below the poverty line, this crisis sparked a nationwide debate about environmental justice—equal treatment and protection from environmental harm regardless of race, ethnicity, or income—and drinking water quality.

Low-income and minority communities often face disproportionate burdens of exposure to contamination sources and environmental pollution, and associations with race and ethnicity persist even after accounting for differences in income [3]. While few studies have looked for links between drinking water and environmental justice indicators (e.g., poverty, race/ethnicity) [4], existing studies have found associations between poorer drinking water quality and these indicators [4–8]. Community water systems (CWSs) that serve communities with lower median incomes, lower rates of home ownership, and higher proportions of Hispanic or non-white residents have been associated with higher levels of nitrate and arsenic [5–7]. Among small rural water systems in Quebec, those serving areas with more material deprivation (based on income, education, and employment) were more likely to have lead levels of health concern and less likely to have advanced water treatment [4]. Health-based violations of the SDWA were more common in poorer communities with higher proportions of Hispanic or African-American residents; the effects of race and ethnicity were not apparent in more affluent communities [8]. Environmental justice associations with drinking water have not been consistently observed, and may depend on the spatial scope and individual contaminants studied. For instance, Cory and Rahman [9] concluded there was limited evidence for environmental justice disparities in exposures to arsenic in water systems in Arizona. The likelihood of studies finding environmental justice associations with the siting of hazardous waste facilities strongly depends on the unit of analysis (e.g., county, census block) and overall scope (e.g., state, national); studies with a small unit of analysis and large scope were most likely to find significant associations [10]. To date, studies of environmental justice and drinking water contaminants have considered individual states (e.g., Arizona, Oregon) or sections of states (e.g., California’s Central Valley); however a nationwide assessment is lacking.

According to Balazs and Ray’s Drinking Water Disparities Framework [11], there are a wide range of natural, built, and sociopolitical factors that can cause and perpetuate disparities in water quality, reliability, and infrastructure. Small water supplies, particularly those that serve low-income and minority communities, may have poorer source water quality due to closer proximity to pollution sources. In addition, such supplies may have diminished technical, managerial, and financial (TMF) capacity to properly manage their drinking water, so these systems may lack the resources necessary to comply with testing requirements. Indeed, a nationwide analysis indicated that small CWSs were more likely to have management-related SDWA violations [12]. When problems are identified, small systems with limited TMF may struggle to address these problems, such as through the installation of new treatment systems or development of better protected sources [1]. In communities of color, institutional barriers in local planning and zoning practices may lead to lower rates of drinking water and wastewater infrastructure improvement [13]. These can relate to both internal factors (diminished ability to raise rates for customers) and external factors (ability to apply for loans). These factors are especially apparent in unincorporated areas, which have no tax base and lie outside of the municipal boundaries overseen by county or state entities.

Nitrate is one of the contaminants most frequently found in violation of health-based standards in U.S. drinking water [14]. Nitrate naturally occurs in aquatic systems at low concentrations (< 1 mg/L NO_3_-N), while concentrations greater than 1 mg/L NO_3_-N are considered elevated above background and indicative of human activity [15]. Common anthropogenic sources of nitrate include fertilizers used for agricultural production and landscaping, animal manure, wastewater discharges from sewage treatment plants and septic systems, and fossil fuel combustion. Elevated levels of nitrate can signal the presence of other contaminants of concern; a study of over 2000 private wells found that wells with > 1 mg/L NO_3_-N were more likely to have levels of pesticides and volatile organic compounds (VOCs) above one-tenth of an MCL or health-based screening level [15]. Among public and private wells in sand and gravel aquifers, nitrate concentrations were correlated with pharmaceuticals and other unregulated drinking water contaminants [16, 17].

As part of the original implementation of the SDWA in 1974, the U.S. EPA established a nitrate MCL of 10 mg/L NO_3_-N (45 mg/L NO_3_^−^) based on case studies of methemoglobinemia in infants who consumed formula mixed with water containing nitrate [18]. More recent epidemiological studies have found associations between nitrate concentrations in drinking water and bladder cancer [19, 20], thyroid cancer [21, 22], colon cancer [23, 24], kidney cancer [25], birth defects [26, 27], low birth weight [28], and preterm birth [29, 30]. Some of these effects were significant for exposures at or above 5 mg/L, particularly over longer exposure periods [21, 23, 26, 27]. The International Agency for Research on Cancer classified “ingested nitrate or nitrite under conditions that result in endogenous nitrosation” as a probable human carcinogen (Group 2A) [31]. Exposure to nitrate in drinking water has also been linked to thyroid dysfunction [32], although the Agency for Toxic Substances and Disease Registry concluded that there is “limited evidence” for nitrate-induced thyroid dysfunction [33]. The U.S. EPA’s Integrated Risk Information System (IRIS) is undertaking a broad re-evaluation of the health effects of nitrate and nitrite [34].

In light of growing epidemiological evidence for nitrate health effects below the MCL and evidence on a local level for socioeconomic disparities in nitrate exposure, our study was designed to evaluate whether nitrate concentrations are elevated in public water supplies that serve communities with higher proportions of low-income and/or minority residents. We hypothesized that CWSs serving communities with higher proportions of Hispanic residents would have higher nitrate levels because 80% of U.S. farmworkers are Hispanic [35] and because synthetic fertilizers used in agriculture are the largest source of nitrogen inputs in the U.S. [36]. We also anticipated that the high cost of removing nitrate from contaminated drinking water would lead to socioeconomic disparities in nitrate exposures. This study represents the first investigation of socioeconomic disparities in drinking water contaminants at the national scale and provides new insights into the interplay of system characteristics and demographic parameters.

## Methods

### Water system and demographic data sources

Detailed information about public water systems was gathered from the U.S. EPA’s Safe Drinking Water Information System (SDWIS) [37]. Our target population were CWSs in each U.S. state that were active at some point between 2010 and 2014. We restricted our analysis to CWSs because these systems serve customers in their homes year-round, whereas non-community systems can serve non-residential settings such as office buildings and campgrounds. We did not include CWSs that purchased their water from another supplier; purchasing water systems are rarely required to test for nitrate and therefore rarely collect nitrate data. In total, we retrieved data for 412,835 systems, of which 42,114 were CWSs active between 2010 and 2014 that did not purchase their water. Relevant characteristics obtained for each system included: activity status, system type (community, non-community, etc.), number of people served, source water type (groundwater or surface water), affiliated wholesaler or purchasing systems, and region served by the system (city, county). CWS system sizes were classified using categories defined by the EPA: very small (≤500 people); small (501–3300); medium (3301–10,000); large (10,001–100,000); and very large (>100,000).

We obtained race, ethnicity, poverty, and home ownership information (2010–2014 five-year estimates) and the proportion of households in urbanized areas (2010 estimates) from the U.S. Census Bureau for each county, census-designated place, and county subdivision in the 50 U.S. states [38, 39]. Agricultural data on the amount of livestock (cows, goats, horses, pigs, sheep) per 100 acres and the percent of land area used as cropland were obtained from the U.S. Department of Agriculture’s 2012 Census of Agriculture for each U.S. county [40]. Demographics and agriculture variables were assumed to remain constant throughout our study period.

### Identifying populations served by CWSs

Characterizing the demographics of the communities served by each water system is challenging in part because little information is publicly available on the geographic areas served by each CWS [41]. Few states provide public access to electronic records documenting the service areas of their public water systems, so we relied on the information included in SDWIS.

SDWIS’s *Water System* module provides address variables conveying the location of each water system’s “legal entity,” i.e., the mailing address of the administrative personnel associated with the system. Separate variables, *city served* and *county served,* describe the areas to which a system directly provides water, and the *primacy agency code* specifies the agency that has regulatory oversight of the water system (typically a state agency encompassing the cities or counties served). Using SDWIS’s *Geographic Area* module, which some states primarily use to report the “areas served” parameters, we were able to augment our database’s cities and counties served.

**Fig. 1:**
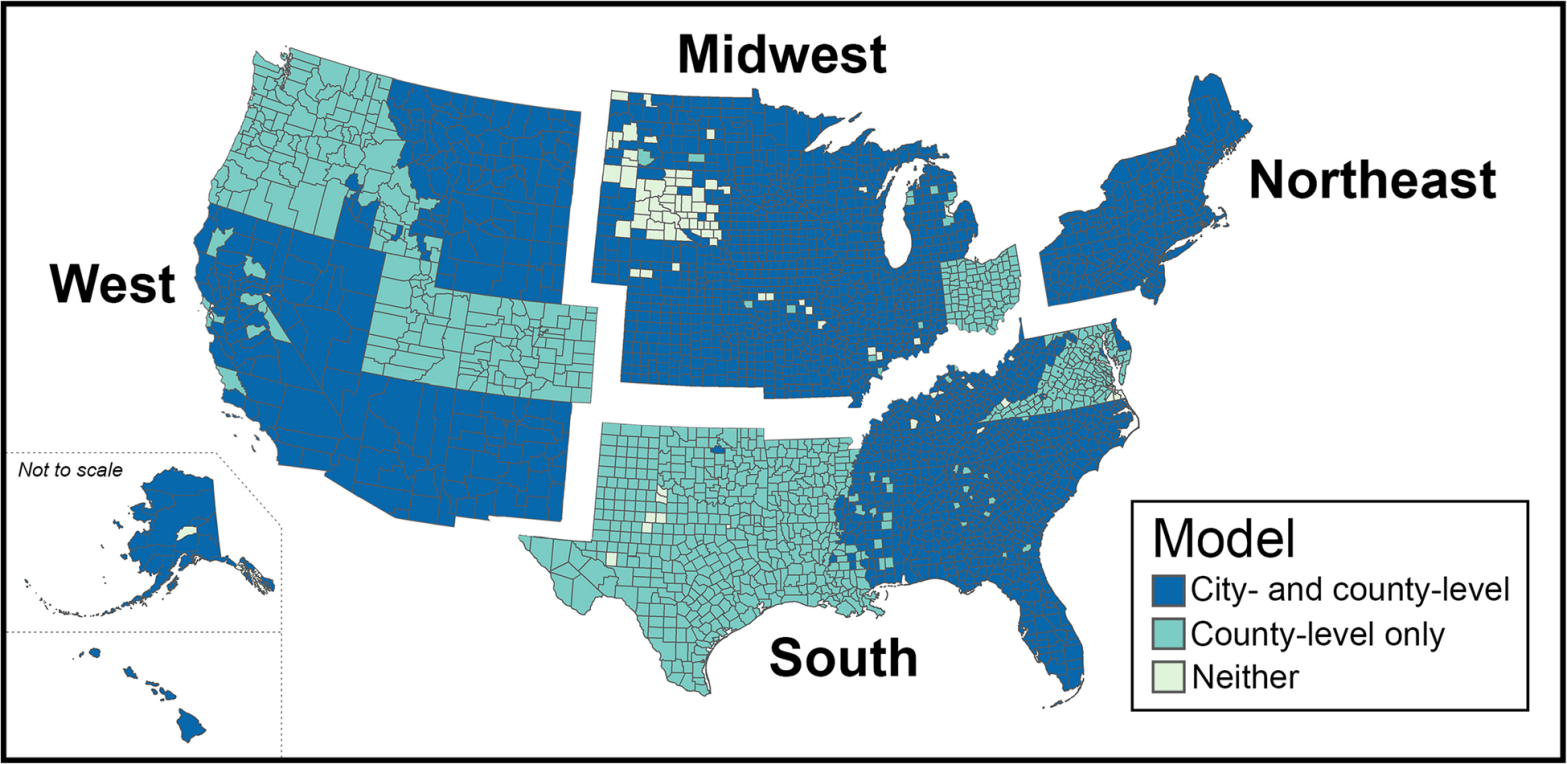
Coverage of the states and regions included in our statistical analyses. Some portions of the country were not included in our analysis either because they did not have a community water system on record or because we were unable to collect nitrate data for that area

We used the *city served* and *county served* fields in SDWIS to determine the areas served by each CWS. SDWIS provided information in the *counties served* field for > 99% of CWSs (*n* = 41,781), but only 48.1% of CWSs reported information in the *cities served* field (*n* = 20,267). By contacting state agencies, we were able to supplement SDWIS data for 1509 CWSs in three states; however, 13 states rarely or never record information in the *cities served* field (Fig. 1). Although administrative address information was available for nearly all water systems, we concluded that such data did not reliably identify the areas served by each CWS. Some system administrator addresses were located hundreds of miles away from the cities served by their affiliated water systems or were located in a different state, and for 40% of the systems with both a *city name* (pertaining to the administrator’s city) and a *city served* designated in SDWIS, the two fields shared no overlapping cities. Furthermore, the demographics of the areas associated with the administrative addresses often varied substantially from the demographics of each water system’s *cities served* (Additional file 1: Table S1).

Data obtained for wholesale water systems varied widely in whether they included the cities or customers served by downstream purchasing systems. Because purchasing systems were not included in our analysis, it was important that the data for each wholesaler included all of the cities that purchase its water in order to best characterize the population served by each CWS. As described in Additional file 1, we amended wholesalers’ city served and population served fields to include the area and people served by systems that purchase wholesale water. As a result of these modifications, we expanded data on the cities served for 1245 wholesale systems (174 of which lacked any city served data prior to considering purchased water systems) and adjusted the system size for 220 wholesale systems. Data that we compiled for U.S. CWSs, including service areas and purchaser-seller connections, are provided in Additional files 2 and 3.

### Linking demographic and water system data

Water system characteristics and demographic data were linked by matching the names in the SDWIS *city served* and *county served* fields with the geographic names in the Census Bureau demographic data. Because the majority of *city served* names matched to county subdivisions in the Northeastern U.S. (Pennsylvania and all states to the north and east), county subdivision demographics were prioritized as a match for CWSs in the Northeast. Census-designated places were prioritized in all other U.S. regions, where county subdivision matching was infrequent. Notably, the *city served* and *county served* fields are not checked for consistency at a national level, so SDWIS entries are prone to local (rather than official) naming conventions and typographical errors. We used an approximate string matching algorithm based on Jaro-Winkler distance to identify the most similar name in the demographic data for each *city served* and *county served* that did not have an identical match. Typographical errors and differences in abbreviation conventions were corrected to improve string matching. All *county served* names provided by SDWIS were matched to Census Bureau records in this manner. For *city served* names that did not match or matched multiple Census Bureau records after these corrections, we gathered additional information from online encyclopedias and search engines about individual water systems and geographical locations. In some cases, this additional information allowed us to definitively link *city served* names from SDWIS with location names in Census Bureau data, while in other cases the names were considered non-matching and were not included in subsequent analyses. In total, we successfully linked demographic data to every CWS that provided a *county served* (41,781 systems) and 96.8% of CWSs for which we had *city served* information (21,253 systems).

For CWSs serving multiple cities or counties, we calculated a weighted average for each demographic parameter based on the relative populations of each of the cities or counties served. For instance, if a CWS served five cities, then the proportion of Hispanic residents associated with that CWS was calculated as an average of the proportion of Hispanic residents in each of the five cities, weighted by each city’s population. When demographic information was missing for one or more cities served, as was the case for 262 systems (1.2%), these weighted averages were calculated based on all available demographic data for cities served by that system.

### Nitrate data

There is no national database of water contaminant concentration data. Thus, nitrate data were acquired by contacting SDWA-designated primacy agencies in each state through a combination of email and Freedom of Information Act requests and by gathering data from ten state-operated online data portals. In total, we compiled data for over 2.7 million samples analyzed for nitrate and/or nitrate-nitrite (sum of nitrate plus nitrite). Of these, 616,591 samples were collected from our CWSs between 2010 and 2014, covering 40,178 (96.2%) of the systems for which we had county-level demographic data.

Nitrate (NO_3_-N) concentrations were used when available to estimate each CWS’s annual nitrate concentration. However, CWSs in 13 states primarily reported nitrate-nitrite (NO_3_/NO_2_-N) data, and some CWSs reported both. In order to extrapolate NO_3_-N concentrations from NO_3_/NO_2_-N concentrations, we used a univariate linear regression based on all samples for which we could pair by system, sampling date, and sub-facility (*n* = 48,925 pairs). This regression ([NO_3_-N] = 0.987*[NO_3_/NO_2_-N], r^2^ ≈ 0.93) was used to estimate NO_3_-N concentrations when only NO_3_/NO_2_-N concentrations were provided.

We made additional adjustments to the dataset to address discrepancies in how nitrate data were reported by states. In some cases units (mg/L, μg/L) were either missing or appeared to be misreported (e.g., 0.2 ng/L), complicating the interpretation of sample results, particularly those that substantially diverged from other samples collected from the same system. We developed a protocol to identify outliers that were either misreported or misevaluated based on the average difference between each sample result and other samples from that system. Outliers were confirmed by visual inspection, and we removed 498 of such cases (0.1% of all samples) from the analysis. In addition, 17 states never provided detection limits (DLs) for samples without detectable nitrate. DLs are useful for quantitative analyses of nondetects to constrain the range of possible values for samples below the DL (e.g., < 0.2 mg/L indicates a more limited range of possible concentrations than < 1 mg/L). Among 151,463 nitrate and nitrate-nitrite samples for which DLs were provided, 99.4% of DLs were ≤ 1 mg/L and 17.4% of DLs were exactly 1 mg/L, so 1 mg/L was used as a reasonable upper-bound DL for all nondetects that were missing a DL (12.3% of all samples).

### Statistical analyses

We used a mixed-effects linear regression model to evaluate the relationship between nitrate concentrations and demographic, system-characteristic, and land-use data. Model parameters were selected a priori to allow us to explore associations between environmental justice indicators and nitrate concentrations while controlling for possible confounders. Environmental justice indicators included those related to race and ethnicity (percent Hispanic residents, percent non-Hispanic Black residents) and those related to poverty (percent of families with income below the poverty line, percent of home ownership). System characteristics of interest included system size and type of source water (surface water or groundwater). Land use variables (number of livestock per 100 acres, percent of land area used as cropland, percentage of homes in urbanized areas) were included to account for agricultural and wastewater contributions to nitrate source water contamination. State was included as a random effect to capture the influence of different monitoring and reporting practices for drinking water and differences in state regulations of pollutant sources and enforcement. In the baseline model, we used county-level demographics because *county served* information was provided for nearly all CWSs, offering nearly complete geographical coverage (Fig. 1). Collinearity was evaluated by visual inspection of Spearman correlation coefficients (Additional file 1: Tables S2-S6) and by calculating variance inflation factors (VIFs) for each model. Race, ethnicity, and at least one poverty indicator were retained in all models, and other covariates were retained if they were significantly associated with the outcome (*p* < 0.05) or if removing them from the model changed the effect estimates for other covariates by more than 10%.

Nitrate data were right skewed and approximately lognormal. Because nitrate concentrations were below the DL in nearly 30% of water samples, we used a multiple imputation method adapted from Lubin et al. [42] to address nondetects. Multiple imputation can provide less biased variance estimates relative to a simple substitution method (typically DL/2 or DL/$\sqrt{2}$) in datasets with more than 10% nondetects [42]. For each year in which a CWS operated (“system-year”), we calculated a minimum average nitrate concentration, imputing zero for nondetects, and a maximum average nitrate concentration, imputing the DL for nondetects. Together, these values constitute an interval that contains every possible system-year average, and for system-years without nondetect data, these two values are identical. Because we lacked information on the relative contributions of multiple sources to overall water flow within a system, we assumed all sub-facilities and all samples contributed equally to the system-year average. For each system, we then calculated a five-year system average concentration as an interval, with the lower/upper bounds of the interval represented as the average of all of the minima/maxima of system-year intervals, respectively. The ranges of these five-year system average intervals were generally narrow: 67.3% of system averages had a range ≤ 0.2 mg/L, 99.8% had a range ≤ 1 mg/L, and 0.2% (91 systems) had a system average interval range > 1 mg/L.

We used non-parametric bootstrap resampling (1000 bootstrap iterations) and maximum likelihood estimation to estimate means ($\overset{\sim }{x}$) and standard deviations ($\overset{\sim }{\sigma }$) of a normal distribution that best fit the natural logarithms of our system average intervals. Natural log-transformed system averages were then randomly imputed in accordance with the proportions of the cumulative distribution function of a normal distribution with mean $\overset{\sim }{x}$ and standard deviation$\overset{\sim }{\sigma }$ truncated at the natural logarithms of the system average minimum and maximum. We did not impute values for system averages based entirely on detected concentrations (37.9% of systems) because we could calculate these averages directly. We then evaluated ln-transformed system averages as the outcome variable in a mixed-effects regression model. This procedure (bootstrap resampling, lognormal parameter estimation, imputation, and regression analysis) was repeated five times to determine the consistency of our results. Regression results from each of these five phases were pooled using techniques outlined by Rubin [43].

To evaluate disparities in drinking water exposures to nitrate levels of health concern, we conducted a separate set of analyses in which we dichotomized the outcome variable. Using the same multiple imputation approach, systems were categorized as “high nitrate” if their estimated system average was ≥5 mg/L, a level associated with adverse health outcomes in some epidemiological studies, and as “low nitrate” otherwise. We used a generalized logistic mixed-effects regression model to evaluate the effects of the same set of predictors, including the state-specific random effect, on the binary nitrate response variable. As before, five sets of regression results were pooled using the Rubin method.

We assessed whether our results depended on geographic region or on the spatial resolution of the demographic data. To evaluate variability across geographic regions, we conducted a similar set of regression analyses stratified into four regions (Midwest, Northeast, South, and West) defined by the U.S. Census Bureau. To evaluate the effect of the spatial scale of the demographic data, we also repeated our analyses using demographic data representing the city or cities served by each CWS. Thirteen states rarely or never report *city served* values in SDWIS (Fig. 1), so a substantial portion (49.4%) of CWSs were not included in this city-level analysis. Since agricultural data were only available at the county level, our analyses using city-level demographics included agricultural variables corresponding to the counties served by each system. Finally, to determine whether differences between the county-level and city-level results were more dependent on the scope (geographic area considered in the analysis) or scale (resolution of the city vs. county demographics), we developed an intermediate third model using county-level demographics for only those CWSs included in our city-level analysis.

All analyses were conducted using R version 3.4.1 [44].

## Results

Of the 42,114 CWSs that did not purchase water from another system and were active between 2010 and 2014, we were able to match 39,466 CWSs to both a complete county-level demographic profile and at least one nitrate measurement from that time period (Additional file 1: Figure S1). The 39,466 CWSs in our analysis served 233.2 million people, more than 70% of the U.S. population (Table 1). Over 90% of the CWSs in our sample served ≤10,000 people (very small, small, and medium size systems); these systems provided water to less than 20% of the population served by public water supplies overall. Nearly 90% of the CWSs in our sample have groundwater sources; however, the majority of the population in our sample area (65%) is served by a CWS with a surface water source.

**Table 1:** Characteristics of community water systems (CWSs) and median demographics of the counties they serve

System characteristics	Study population		Nitrate characteristics		Median county demographic characteristics			
	Number of CWSs	Population served	CWSs ≥5 mg/L NO_3_-N (%)	95th percentile [NO_3_-N] (mg/L)	Percent Black, non-Hispanic (Q1, Q3)	Percent Hispanic (Q1, Q3)	Percent below poverty line (Q1, Q3)	Percent home ownership (Q1, Q3)
Overall	39,466	233,153,669	4.2	4.60	3.0 (0.8, 11.2)	6.0 (2.6, 13.1)	15.0 (11.5, 18.6)	70.9 (65.1, 75.9)
System Size								
V. Small	23,198	3,624,068	4.8	4.89	2.6 (0.8, 9.6)	6.4 (2.8, 13.2)	14.7 (11.3, 18.3)	70.3 (64.9, 75.6)
Small	9867	13,934,810	3.7	4.30	3.2 (0.8, 12.8)	4.8 (2.1, 11.8)	15.6 (11.9, 19.5)	72.5 (67.2, 76.8)
Medium	3437	20,032,495	2.7	3.78	4.2 (1.1, 14.3)	4.9 (2.3, 12.6)	16.1 (12.2, 19.6)	71.4 (65.7, 76.0)
Large	2614	75,884,314	2.9	3.92	5.0 (1.8, 11.6)	6.8 (3.1, 15.7)	15.3 (11.6, 18.8)	69.4 (63.4, 74.3)
V. Large	350	119,677,982	3.1	4.03	9.6 (4.9, 17.6)	13.4 (6.4, 27.7)	15.5 (12.5, 18.4)	63.4 (57.7, 68.1)
Region								
Midwest	8869	42,146,514	5.5	5.20	1.2 (0.5, 4.0)	2.9 (1.7, 5.3)	13.4 (10.8, 16.7)	74.5 (69.8, 78.0)
Northeast	6572	31,734,958	3.5	4.34	2.6 (1.0, 5.1)	3.8 (1.8, 9.3)	11.6 (9.1, 14.2)	71.7 (68.3, 76.2)
South	14,452	94,026,660	2.4	3.34	14.6 (6.0, 25.7)	6.1 (3.2, 14.1)	18.0 (13.7, 21.4)	71.8 (64.9, 75.9)
West	9573	65,245,537	6.1	5.42	1.0 (0.5, 2.6)	12.7 (7.8, 30.6)	16.0 (12.3, 19.3)	65.7 (60.1, 70.2)
Source Water								
Groundwater	35,215	80,860,916	4.5	4.78	3.2 (0.9, 12.1)	6.1 (2.6, 13.0)	14.9 (11.4, 18.5)	71.0 (65.1, 76.0)
Surface water	4251	152,292,753	1.2	2.64	2.3 (0.8, 6.7)	5.5 (2.4, 13.1)	15.8 (12.3, 19.3)	70.6 (65.2, 75.1)

The majority of estimated annual mean nitrate concentrations were below 1 mg/L for all system sizes, regions, and source water types. However, 1647 CWSs, serving over 5.6 million Americans, had average nitrate concentrations at or above 5 mg/L from 2010 to 2014. The West and Midwest had the highest proportions of high nitrate systems (≥5 mg/L) and the highest 95th percentile nitrate concentrations, while the South had the lowest (Table 1). The proportion of high nitrate CWSs was nearly four times higher for systems that rely on groundwater sources compared to CWSs that rely on surface water sources, and the 95th percentile concentration for groundwater systems was more than 2 mg/L higher. Relative to other system sizes, very small systems (≤500 people) had the highest 95th percentile concentration and the highest proportion of high nitrate systems.

Among environmental justice indicators, race and ethnicity differed among regions and across system sizes, while wealth-related factors were less variable. Black and Hispanic residents made up a larger proportion of residents served by very large systems than smaller systems. Systems in the South served the highest proportion of Black residents, and CWSs in the West served the highest proportion of Hispanic residents. The proportion of residents with annual incomes below the U.S. Census poverty threshold did not vary considerably as a function of system size, while rates of home ownership were lowest in counties served by very large systems. Poverty rates were highest in the South and West, and rates of home ownership were lowest in the West.

We observed significant differences in the demographics and land use patterns between high nitrate and low nitrate (mean < 5 mg/L) CWSs (Table 2). High nitrate CWSs served nearly twice as many Hispanic residents on average compared to low nitrate CWSs, and CWSs that served the highest proportion of Hispanic residents (top quartile) exceeded 5 mg/L nitrate nearly three times as often as CWSs serving the lowest proportion of Hispanic residents (lowest quartile; Additional file 1: Figure S2). By contrast, high nitrate CWSs served less than half as many Black residents on average compared to low nitrate CWSs. Rates of poverty and home ownership were marginally, albeit significantly, lower in counties served by systems with higher nitrate concentrations. Counties with the most agricultural and livestock production had higher proportions of high nitrate CWSs than counties with less agricultural and livestock production (Additional file 1: Figure S2). For instance, 9.1% of CWSs were high nitrate in counties in the top quartile for cropland, while only 1.8% of CWSs were high nitrate in counties in the bottom quartile for cropland.

**Table 2:** Mean county-level demographic and land-use characteristics in low and high nitrate community water systems

Variable	All systems (*n* = 39,466)	Low nitrate CWSs^a^ (*n* = 37,819)	High nitrate CWSs^b^ (*n* = 1647)
Percent Black, non-Hispanic (%)	8.4	8.6	4.0^#^
Percent Hispanic (%)	11.4	11.1	18.1^#^
Percent poverty (%)	15.7	15.7	14.8^#^
Percent home ownership (%)	70.1	70.2	68.6^#^
Percent urban households (%)	36.5	36.3	40.3^#^
Percent cropland (%)	20.2	19.6	35.5^#^
Livestock (number per 100 acres)	9.5	9.2	16.6^#^

Note: Means calculated by imputing zero for nondetects

^a^Mean nitrate < 5 mg/L NO_3_-N

^b^Mean nitrate ≥ 5 mg/L NO_3_-N

^#^Mean significantly different between two groups (high vs. low nitrate) using unequal variances t-test, (*p* < 0.001)

In our national mixed-effects regression analyses using county-level demographic data, race and ethnicity variables showed similar associations with both system average nitrate concentrations and the likelihood of system averages exceeding 5 mg/L (Table 3). A one-unit increase in the percent of Hispanic residents was associated with a 1.8% increase in nitrate concentrations [95% confidence interval (CI): 1.6, 2.0%] and a 1.9% increase in the likelihood of high nitrate concentrations (95% CI: 1.4, 2.4%). By contrast, a one-unit increase in the percent of non-Hispanic Black residents was associated with a 1.3% decrease in nitrate concentrations (95% CI: –1.5, −1.0%) and a 4.3% decrease in the likelihood of high nitrate (95% CI: –5.7, −2.9%). Similar effect estimates were observed in unadjusted models (Additional file 1: Table S7). Poverty, but not home ownership, was inversely associated with nitrate in the adjusted models, with a one-unit increase in the percent of people with incomes below the poverty line associated with 0.8% lower nitrate concentrations (95% CI: –1.2, −0.3%) and a 2.2% decrease in the likelihood of high nitrate (95% CI: –3.7, −0.7%). In unadjusted models, we observed a similar estimate for the association between poverty and nitrate concentrations but did not observe an association between poverty and likelihood of high nitrate, and in contrast to the adjusted models, home ownership showed significant inverse associations with both outcomes (Additional file 1: Table S7).

**Table 3:** Associations between nitrate in community water systems and demographic, land use, and water system characteristics

Variable	Nitrate concentration		Likelihood of high nitrate^a^	
	Percent change (95% CI)	*p*-value	Percent change (95% CI)	*p*-value
Percent Black, non-Hispanic^b^	−1.3 (−1.5, −1.0)	< 0.0001	−4.3 (−5.7, −2.9)	< 0.0001
Percent Hispanic^b^	1.8 (1.6, 2.0)	< 0.0001	1.9 (1.4, 2.4)	< 0.0001
Percent poverty^b^	−0.8 (−1.2, −0.3)	0.0004	−2.2 (−3.7, −0.7)	0.004
Percent home ownership^b^	--^f^		−0.7 (−1.9, 0.4)	0.214
Percent urban households^b^	0.0 (0.0, 0.1)	0.14	0.4 (0.2, 0.6)	< 0.0001
System size: Small^c^	9.0 (4.6, 13.7)	< 0.0001	−21.8 (−31.6, −10.5)	0.0003
System size: Medium^c^	15.8 (9.2, 22.8)	< 0.0001	−24.8 (−40.4, −5.1)	0.016
System size: Large^c^	36.2 (27.1, 45.9)	< 0.0001	−10.0 (−30.6, 16.7)	0.426
System size: V. Large^c^	51.0 (28.2, 77.9)	< 0.0001	54.1 (−20.4, 199)	0.200
Source water: Groundwater^d^	33.9 (26.4, 41.9)	< 0.0001	311 (201, 460)	< 0.0001
Percent cropland^b^	0.6 (0.5, 0.8)	< 0.0001	3.3 (3.0, 3.7)	< 0.0001
Livestock per 100 acres^b^	0.1 (0.0, 0.2)	0.008	0.2 (0.0, 0.5)	0.107
Region: Northeast^e^	84.0 (6.0, 219)	0.030	69.1 (−41.7, 390)	0.333
Region: South^e^	58.5 (−1.7, 156)	0.059	106 (−18.5, 418)	0.127
Region: West^e^	88.1 (13.9, 211)	0.014	270 (44.7, 849)	0.006

Notes: Percent change in nitrate concentration and likelihood of exceeding 5 mg/L associated with a one-unit increase in the county-level demographic data (e.g., 1% increase in percent Hispanic) and agricultural land use data, and for each system size, region, and source water categories relative to the referent group

*N* = 39,466. Models include state-specific random intercepts and a fixed intercept term

^a^Logistic regression; outcome coded as “1” if system-average concentration ≥ 5 mg/L and “0” otherwise

^b^Continuous predictor, table estimates reflect effect of one-unit change in the predictor

^c^Referent group: very small systems. ^d^Referent group: surface water. ^e^Referent group: Midwest

^f^Not included in final model

We observed complex relationships between nitrate levels and system size. In the binary model, very small systems had a greater likelihood of high nitrate compared to small and medium systems. By contrast, very small systems were predicted to have lower concentrations than other system sizes when nitrate was evaluated as a continuous variable (compared to very small systems, nitrate concentrations were 9.0% higher in small systems and 51% higher in very large systems; Table 3). In unadjusted models, the same observations were noted; very small systems were predicted to have greater likelihood of high nitrate but lower continuous nitrate concentrations than all other system sizes (Additional file 1: Table S7). For other variables related to water system characteristics and land use, systems relying on groundwater sources had 34% higher nitrate concentrations compared to systems relying on surface water (95% CI: 26, 42%) and were more than four times as likely to have high levels of nitrate (odds ratio = 4.1; 95% CI: 3.0, 5.6). The extent of cropland coverage and livestock production had significant but small associations with nitrate concentrations, while a one-unit increase in the percent of land area used as cropland had a more substantial effect (3.3% increase, 95% CI: 3.0, 3.7%) on the likelihood that a CWS had high nitrate.

The results of our analysis using city-level demographics, based on the subset of CWSs that provided city served information (50.6%, Fig. 1), varied in several notable ways from the results of our broader county-level analysis. In the city-level analysis, poverty was not associated with nitrate concentrations (Table 4), while home ownership, which was not significant in the national county-level analysis, was associated with lower nitrate. A one unit increase in percent home ownership (roughly equivalent to a one unit decrease in percent renters) was predicted to result in 0.4% lower nitrate (95% CI; −0.6, −0.2%). Similarly, the association between urbanicity and nitrate was significant in the city-level, but not the county-level analysis; a one-unit increase in the percent of households located in urbanized areas was associated with a 0.2% increase in nitrate (95% CI: 0.1, 0.3%).

**Table 4:** Comparison of regression results for nitrate in community water systems using city- and county-level demographics

Variable	City Demographics		County Demographics	
	Percent change (95% CI)	*p*-value	Percent change (95% CI)	*p*-value
Percent Black, non-Hispanic^a^	−0.4 (−0.7, −0.2)	0.0016	−0.7 (−1.1, −0.3)	0.001
Percent Hispanic^a^	0.3 (0.1, 0.5)	0.017	0.9 (0.6, 1.3)	< 0.0001
Percent poverty^a^	−0.2 (−0.6, 0.1)	0.170	−1.9 (−2.5, −1.2)	< 0.0001
Percent home ownership^a^	−0.4 (−0.6, −0.2)	0.0004	0.3 (−0.3, 0.8)	0.325
Percent urban households^a^	0.2 (0.1, 0.3)	< 0.0001	0.1 (0.0, 0.2)	0.222
System size: Small^b^	11.8 (5.5, 18.5)	0.0002	11.4 (5.2, 18.0)	0.0002
System size: Medium^b^	12.3 (3.8, 21.5)	0.004	14.1 (5.6, 23.3)	0.0009
System size: Large^b^	22.8 (12.2, 34.4)	< 0.0001	29.7 (18.6, 41.8)	< 0.0001
System size: V. Large^b^	21.2 (−2.5, 50.5)	0.083	34.7 (8.7, 67.1)	0.007
Source water: Groundwater^c^	35.8 (24.9, 47.7)	< 0.0001	35.2 (24.4, 47.0)	< 0.0001
Percent cropland^a^	0.5 (0.3, 0.6)	< 0.0001	0.4 (0.2, 0.6)	< 0.0001
Livestock per 100 acres^a^	0.1 (0.0, 0.2)	0.123	0.1 (−0.1, 0.2)	0.313
Region: Northeast^d^	62.3 (−8.8, 189)	0.100	−13.8 (−52.2, 55.5)	0.622
Region: South^d^	48.2 (−13.8, 155)	0.155	−3.2 (−44.7, 69.6)	0.910
Region: West^d^	82.1 (2.4, 224)	0.041	−42.9 (−67.5, 0.2)	0.051

Notes: Percent change in nitrate concentration from pooled regression results using city- and county-level demographic, land use, and water system characteristics. Analyses based only on CWSs with available information about both the cities and counties served (*N* = 19,987). Models include state-specific random intercepts and a fixed intercept term

^a^Continuous predictor, table estimates reflect effect of one-unit change in the predictor

^b^Referent group: very small systems. ^c^Referent group: surface water. ^d^Referent group: Midwest

To evaluate whether differences between the city- and county-level analyses were related to the refined spatial scale of cities or trends specific to the portion of the country that provided city information, we conducted an additional analysis using county-level demographics for only those CWSs that provided city served information (“county-level subset”). Overall, the results of this county-level subset model were similar to the results of the nationwide analysis using county-level data. Although the magnitude of some coefficients in the county-level subset analysis changed relative to the national analysis (for instance, race/ethnicity had approximately half of the effect on nitrate concentrations and poverty had about twice the effect), the statistical significance and directionality of the predictors were comparable (Table 4). The results of this comparison suggest that the differences between the county- and city-level analyses are primarily due to different relationships between nitrate levels and demographic predictors at various spatial scales, rather than being an artifact of the part of the country evaluated in the subset analyses.

We included region as a covariate in our nationwide models because of regional differences in nitrate concentrations and demographic characteristics (Table 1) and significant differences among some regions in unadjusted models (Additional file 1: Table S7). In the unadjusted models, the Midwest and West had the highest proportions of high nitrate systems, while the West had higher nitrate concentrations than the Midwest and South. In the adjusted models, the Midwest had significantly lower nitrate concentrations than other regions, while only the West had a higher proportion of high nitrate systems than the Midwest. To investigate potential regional differences in associations among demographics, water system characteristics, land use, and nitrate concentrations, we stratified our nationwide model by region.

In these regionally stratified models using county-level demographics, no single feature had the same impact on system average nitrate concentrations across all four U.S. regions (Table 5). The Midwest was the only region in which the percent of cropland was not associated with nitrate and the only region in which percent of Hispanic residents did not have a positive association with nitrate levels. The Midwest also had the strongest effect of urbanicity; a one-unit increase in the percent of urban households was associated with 0.4% lower nitrate (95% CI: –0.6, −0.3%). The percent of non-Hispanic Black residents was only significantly associated with nitrate in the South, where a one-unit increase was associated with a 1.2% reduction in nitrate levels (95% CI: –1.5, −1.0%), nearly the same as for the U.S. as a whole (1.3% reduction). Home ownership had a strong inverse association with nitrate in the West, where a one unit increase in percent home ownership was associated with a 1.4% decline in nitrate concentrations (95% CI: –2.0, −0.7%); in the other three regions, home ownership was not associated with nitrate. The effect of groundwater source water on nitrate concentrations varied substantially across the regions: in the West, systems with groundwater sources had 139% higher levels of nitrate than those with surface water sources (95% CI: 115, 167%), while in the South, systems that relied on groundwater had 17% lower nitrate than systems with surface water sources (95% CI: –24, −8.1%). Nitrate concentrations increased with system size in the Midwest and West, with very large systems in the Midwest having the largest effect (223% increase relative to very small systems, 95% CI: 102, 418%).

**Table 5:** Regression results for nitrate in community water systems stratified by region

Variable	Midwest^a^ (*n* = 8869)		Northeast^a^ (*n* = 6572)		South^a^ (*n* = 14,452)		West^a^ (*n* = 9573)	
	Percent Change (95% CI)	*p*-value	Percent Change (95% CI)	*p*-value	Percent Change (95% CI)	*p*-value	Percent Change (95% CI)	*p*-value
Percent Black, non-Hispanic^b^	−0.4 (−1.6, 0.7)	0.481	−1.1 (−2.4, 0.2)	0.101	−1.2 (−1.5, −1.0)	< 0.0001	1.0 (−0.9, 2.9)	0.289
Percent Hispanic^b^	−0.8 (−1.5, 0.0)	0.045	4.0 (3.1, 5.0)	< 0.0001	1.9 (1.6, 2.2)	< 0.0001	1.4 (1.1, 1.7)	< 0.0001
Percent poverty^b^	0.8 (−0.4, 2.0)	0.181	−3.8 (−5.0, −2.6)	< 0.0001	−0.6 (−1.2, 0.1)	0.116	−1.7 (−2.4, −1.0)	< 0.0001
Percent home ownership^b^	0.4 (−0.4, 1.2)	0.331	--^e^		0.5 (0.0, 1.0)	0.063	−1.3 (−1.9, −0.7)	< 0.0001
Percent urban households^b^	−0.4 (−0.6, −0.3)	< 0.0001	0.2 (0.1, 0.4)	0.003	0.1 (0.0, 0.2)	0.083	--^e^	
System size: Small^c^	33.0 (23.2, 43.6)	< 0.0001	30.4 (18.2, 44.0)	< 0.0001	−8.9 (−15.0, −2.5)	0.011	3.5 (−4.5, 12.3)	0.402
System size: Medium^c^	54.4 (36.6, 74.5)	< 0.0001	27.3 (9.6, 48.0)	0.002	−14.8 (−21.8, −7.2)	0.0003	18.5 (3.9, 35.2)	0.012
System size: Large^c^	103 (74.1, 137)	< 0.0001	35.9 (13.5, 62.6)	0.0008	−11.2 (−20.5, −0.9)	0.036	38.8 (21.0, 59.1)	< 0.0001
System size: V. Large^c^	223 (102, 418)	< 0.0001	23.1 (−18.5, 85.9)	0.324	−14.8 (−33.6, 9.3)	0.208	98.6 (47.3, 168)	< 0.0001
Source water: Groundwater^d^	−14.8 (−27.3, −0.1)	0.049	53.3 (34.7, 74.4)	< 0.0001	−16.6 (−24.3, −8.1)	0.0003	139 (115, 167)	< 0.0001
Percent cropland^b^	−0.2 (−0.3, 0.0)	0.100	1.4 (0.8, 2.1)	< 0.0001	1.4 (1.2, 1.6)	< 0.0001	0.5 (0.2, 0.7)	0.0006
Livestock per 100 acres^b^	0.1 (−0.1, 0.2)	0.304	0.7 (0.3, 1.1)	0.0005	−0.1 (−0.3, 0.0)	0.085	1.7 (1.2, 2.2)	< 0.0001

Note: Percent change in nitrate concentration from pooled regression results using county-level demographic, land use, and water system characteristics in each of the four U.S. regions. Models include state-specific random intercepts and a fixed intercept term

^a^For a visual depiction of which states are in each U.S. region, refer to Fig. 1

^b^Continuous predictor, table estimates reflect effect of one-unit change in the predictor

^c^Referent group: very small systems. ^d^Referent group: surface water. ^e^Not included in final model

## Discussion

This study represents the first nationwide analysis of socioeconomic disparities in exposures to contaminants in public drinking water. We found that 5.6 million Americans relied on a public water supply with an average nitrate concentration ≥ 5 mg/L, one-half of U.S. EPA’s drinking water standard, over the five-year period spanning 2010–2014. Epidemiological studies have suggested that long-term exposure to water with nitrate concentrations above 5 mg/L may be associated with some types of cancer, birth defects, and preterm birth [19, 23, 27, 29]. We found that the proportion of Hispanic residents was significantly associated with nitrate levels, while the proportion of Black residents was inversely associated with nitrate levels. The associations with poverty and home ownership were mixed; when we used the demographics of the counties served by each water supply, we found that poverty was negatively associated with nitrate, while home ownership, an indicator of wealth and political empowerment, was inversely associated with nitrate when we used city-level demographics. Very small water systems (serving ≤ 500 people) had the highest nitrate levels overall, but after adjusting for demographics and local land use (cropland, livestock production, and urbanicity), very small systems were predicted to have lower nitrate levels than larger systems.

We found that the percent of Hispanic residents was associated with higher nitrate levels in our nationwide analysis and in all U.S. regions except the Midwest. These associations were modest; nationally, a 10% increase in the proportion of Hispanic residents (i.e., increasing from 10 to 20%) served by a CWS was associated with a 19.6% increase in nitrate concentration. Balazs et al. [6] also saw an association between percent Hispanic residents and nitrate levels in small public water supplies in California’s Central Valley, an agriculturally intensive area. We had hypothesized that proportion of Hispanic residents would be associated with nitrate because many agricultural communities have a high proportion of Hispanic residents. However, our observed association persisted even after we adjusted for agricultural activity by including cropland and livestock production as covariates in our models (Table 3) and our nationwide correlation analysis found a negative correlation between the proportion of Hispanic residents and percent cropland (Additional file 1: Table S2). These results suggest that the association between Hispanic residents and nitrate is not solely explained by proximity to agricultural sources, although adjusting for county-level cropland and livestock production will not eliminate residual confounding of the association between proportion of Hispanic residents and nitrate concentration by agricultural contamination of source waters since our covariates do not account for other factors such as soil type, rates of fertilizer use, and adoption of best management practices to control fertilizer runoff. Nevertheless, while agriculture is the largest source of land-based nitrogen inputs, major sources are also present in urban areas, including wastewater treatment plants, leaking sewer lines, and urban runoff [14]. Our correlation analysis found a positive correlation between percent Hispanic residents and percent urban households (Additional file 1: Table S2), suggesting that some of the association between Hispanic residents and nitrate levels may be related to nitrate sources in urban areas.

In addition to proximity to nitrate pollution sources, the observed relationship between proportion of Hispanic residents and nitrate may be indicative of disparities in TMF resources related to source water protection and water treatment. Communities with higher proportions of minority residents, particularly those who are non-native English speakers, may have less political influence and may be disenfranchised from political and budgetary decision-making processes [11], and therefore may have fewer resources to install new treatment technology or develop new source waters in response to contamination. In our analysis, we are not able to identify the relative importance of proximity to nitrate sources and management-related factors. Nevertheless, our findings are consistent with prior studies in which Hispanic communities were found to have higher drinking water exposures to arsenic, another contaminant regulated under the SDWA. In Oregon, communities served by CWSs in violation of the arsenic MCL had a much higher proportion of Hispanic residents [5], and in Arizona, the proportion of Hispanic residents served by a public water system was positively associated with the likelihood that that system violated the arsenic MCL [9]. Since arsenic in groundwater often comes from geogenic rather than anthropogenic sources [5, 7], these associations may be indicative of disparities in the ability of communities to afford enhanced drinking water treatment technologies, and taken together, suggest that Hispanic communities may experience elevated exposures to multiple drinking water contaminants.

In contrast to our results for Hispanic residents, we observed that the proportion of Black residents was inversely associated with nitrate on a national level, although this association was only observed in the South in our regional analysis. The inverse association observed nationally may be heavily influenced by the South, which has the lowest proportion of high nitrate systems, the most water systems, and the highest proportion of Black residents of all regions. In the South, the negative relationship between the proportion of Black residents and nitrate levels may be explained in part by biogeochemical factors. Pennino et al. [14] suggested that the lack of nitrate MCL violations in Louisiana, Mississippi, and Alabama—states that all have > 25% Black residents—may be associated with biological uptake and transformation processes and regional geological factors. The finding of no significant association between the proportion of Black residents and nitrate levels in the West is consistent with findings of Balazs et al. [6], who observed no significant relationship between non-Hispanic people of color and nitrate levels in California’s Central Valley.

The associations between wealth-related parameters (poverty and home ownership) and nitrate levels differed among U.S. regions and various spatial resolutions of the demographic data. In our national adjusted models using county-level demographics, poverty was inversely associated with nitrate levels while home ownership was not associated with nitrate. By contrast, when we used demographic data corresponding to cities and towns rather than counties, we observed that poverty was not associated with nitrate and that home ownership was inversely associated with nitrate, implying that cities and towns with higher proportions of renters tend to have higher nitrate levels. The effect estimates for poverty and home ownership varied between unadjusted and adjusted models, although our model building approach with a priori variable selection does not indicate which covariates were associated with the most substantial changes in these estimates. As with race/ethnicity, wealth can be expected to relate to levels of contamination in two ways: proximity to pollution sources and ability to treat contaminated source water. While we adjusted our models for agriculture and urbanicity, these variables may not have captured proximity to other important sources of nitrate inputs, such as landfills, industrial facilities, fossil fuel combustion, and home building [36, 45]. Such factors may be related to wealth to the extent that they are driven by economic activity. Discrepancies between our city- and county-level analyses could be explained if county-level wealth operated in our model as the best proxy for nitrogen sources not accounted for by other variables, while city-level wealth better represented civic engagement, capacity to raise customer rates, and, by consequence, ability to treat contaminated water.

We hypothesized that smaller water systems would have higher nitrate concentrations. Smaller water systems may have fewer financial and technical resources to address contamination issues when they arise [1], and the cost of water treatment per household is considerably higher for smaller systems because of a lack of economies of scale [9]. Indeed, very small systems had higher 95th percentile nitrate concentrations than larger systems and were more likely to exceed 5 mg/L nitrate in unadjusted models. Additionally, after adjusting for demographic and land use parameters, very small systems were more likely to have high nitrate compared to small and medium sized systems. However, in our regression analyses with nitrate as a continuous variable, very small systems had lower nitrate concentrations compared to larger system size categories nationwide (Table 3) and in the Midwest, Northeast, and West (Table 5). Taken together, these results suggest that larger systems have higher nitrate on average, but that very small systems are more likely than other system sizes to have nitrate concentrations at the high end of the distribution. Previous studies have found inconsistent relationships between system size and contaminant violations. Switzer and Teodoro [8] identified a negative relationship between the population served by a system and the system’s number of health-based SDWA violations (MCL and treatment technique violations) in a subset of CWSs across the U.S., while Rahman et al. [46] reported a positive association between MCL violations and the number of people served by water systems in Arizona. In a purely statistical sense, larger water systems may be more likely to detect elevated nitrate levels because they are required to test more frequently and, in the case of groundwater systems, might draw from a greater number of source water wells.

Beyond health-based violations, very small CWSs were reported to have more frequent violations of monitoring and reporting requirements than larger systems [47]. This observation is consistent with our data: of the 41,781 CWSs we paired with county demographics, very small systems were significantly more likely than larger systems to lack nitrate sample results over the five-year study period (5.2% of very small systems missing nitrate data compared to 1.8% of larger systems; Pearson χ^2^ = 301). This difference is unlikely to be due to differences in testing requirements, since CWSs are required to test for nitrate annually or more frequently [48]. Such difficulties in adequately monitoring drinking water contaminants likely stem from limited financial resources and/or managerial expertise, and may signal concurrent challenges in conforming to SDWA health-based guidelines. In this regard, Balazs and Ray [11] reported that very small water systems in Fresno County, California, that had failed to monitor for drinking water contaminants under county governance were found to have MCL violations when state officials investigated.

Strengths of our study include the extensive scale and completeness of our dataset for both demographics and water quality data, and our use of information about purchasing water systems to link water quality data with entire areas served by CWSs. A major limitation to our analysis is the potential for exposure misclassification. Because we lacked information about flow volumes from multiple sources within CWSs, we weighted all samples collected for each CWS equally. In some cases, this may have led to overestimates of nitrate concentrations in systems where more contaminated sources are pumped less frequently or only maintained for backup; this overestimation may affect groundwater systems more since we anticipate that they may have more intake points than CWSs with surface water sources. Furthermore, nitrate concentrations may show substantial intra-annual variability, so depending on when nitrate samples were collected within each year, our aggregated metrics may not capture the true average nitrate concentration for each CWS. As with any study of population-level data, we are limited in our ability to draw conclusions about individual exposures and thus limited in our ability to infer causal relationships between the EJ variables of interest and exposure to nitrate in drinking water. Nitrate levels in CWSs may not accurately reflect the exposures among residents in those areas because some residents may rely on bottled water. Use of bottled water may exacerbate disparities in pollutant exposures because lower-income residents have lower ability to pay for bottled water. Another limitation was that we lacked information about cities and towns served for about half of the CWSs in our analysis and so we had to rely on county-level demographics in most analyses, which is a limitation because demographic data for a county may not accurately reflect the demographics of all cities and towns within each county. Our analysis using city-level demographics is limited in its geographic scope, although this did not seem to account for the differences in model results using city- and county-level demographic data. Ideally, we would use census block level information to provide the best resolution of demographic data. However, because geocoded information that specifies the CWS serving each census block is not available, we could not analyze data at the census block level. For small CWSs that serve part of a large city, we used the demographics of the whole city, but the demographics of the population served by the CWS may vary from those of the city overall. Some CWSs were not included in our analysis because we could not match the names of the cities and towns served with locations in the Census Bureau data, although this accounted for a very small proportion of systems. We also could not include unincorporated areas in our city-level analysis because they are not included in Census Bureau data. Finally, we were unable to compile nitrate data for all CWSs. In some cases, this was related to data handling problems; for instance, some records were only available in paper reports, and in other cases, this may reflect a lack of compliance with monitoring requirements, which is more likely for small rural communities who may not be able to afford testing or where there is less enforcement of testing requirements.

Our study did not include the 44 million Americans who rely on a private well for their drinking water, for whom water quality testing is not required under the SDWA. Private wells are shallower than public wells, and shallow wells are more vulnerable to nitrate contamination [49]. They are also more likely to be located in rural areas and may be in closer proximity to agriculture and livestock production sources. Private well owners are usually not required to test for nitrate or other drinking water contaminants, so their presence may go undetected. Among nearly 4000 private wells tested in rural Wisconsin by a state water quality laboratory, nearly 10% exceeded the nitrate MCL [50]. There is evidence of environmental justice disparities in communities using private wells or lacking piped-water entirely. A review by VanDerslice [41] summarized case studies of minority communities reliant on contaminated private wells. For instance, in a low-income Hispanic community of 25,000 in the Yakima Valley in Washington State, more than 10% of private wells exceeded the nitrate MCL [51]. These case studies are further evidence of impaired water quality in communities reliant on private wells and indicate potential socioeconomic disparities in these communities as well.

## Conclusions

This study represents the first nationwide analysis of socioeconomic disparities in exposures to drinking water contaminants, and the framework that we developed in this study can be extended to investigate disparities in exposures to other drinking water contaminants. We found that communities with higher proportions of Hispanic residents tend to be served by community water systems with higher nitrate and greater likelihood of being over 5 mg/L. Our regression analyses indicate that this association is not completely explained by proximity to cropland and livestock production. While > 99% of CWSs do not exceed the nitrate MCL of 10 mg/L, 5.6 million Americans are served by CWSs with nitrate concentrations above 5 mg/L. Nitrate data for private wells, which are even more vulnerable to nitrate contamination, are lacking. Understanding the extent of current exposures, particularly among vulnerable subpopulations, is critical for developing effective strategies to reduce exposures in these communities. Our findings suggest that programs intended to help low-income and small CWSs may not be adequately assisting communities with high proportions of Hispanic residents. Epidemiological evidence for adverse health effects associated with consumption of drinking water above 5 mg/L nitrate raise concerns about increased risk in people exposed at this level and support a re-evaluation of the federal nitrate standard. Even well below the standard, nitrate levels of 1 mg/L or higher are associated with anthropogenic impact; thus nitrate may be an inexpensive indicator to identify drinking water systems that may also contain other contaminants of concern.

## Additional files

Additional file 1: Supplemental description of methods and additional tables and figures. **Table S1** Number of systems with discrepancies between demographics characterized using SDWIS’s *City served* and*Zip code* fields. **Table S2** Spearman correlation matrix for county-level predictors, all systems. **Table S3** Spearman correlation matrix for county-level predictors, Midwest systems. **Table S4** Spearman correlation matrix for county-level predictors, Northeast systems. **Table S5** Spearman correlation matrix for county-level predictors, South systems. **Table S6** Spearman correlation matrix for county-level predictors, West systems. **Table S7** Unadjusted univariate regression model results using nationwide data and county-level demographics. **Figure S1** Number of community water systems compiled from SDWIS and subsequently used for regression analyses. **Figure S2** Percent of CWSs with mean nitrate ≥5 mg/L by quartile for land use and demographic variables. (PDF 13,659 kb)

Additional file 2: Data for U.S. community water systems, including service areas and purchaser-seller connections. (PDF 6544 kb)

Additional file 3: Data glossary for U.S. community water systems. (PDF 1866 kb)

## Abbreviations

CWS: Community water system
DL: Detection limit
EPA: United States Environmental Protection Agency
MCL: Maximum contaminant level
MWRA: Massachusetts Water Resources Authority
NO_3_-N: Nitrate nitrogen
SDWA: Safe Drinking Water Act
SDWIS: Safe Drinking Water Information System
TMF: Technical, managerial, and financial capacity
