# Supplementary material for: Environmental justice and drinking water quality: are there socioeconomic disparities in nitrate levels in U.S. drinking water?
Source: Environ Health. 2019 Jan 17;18:3. doi: 10.1186/s12940-018-0442-6 (PMC6335696; doi:10.1186/s12940-018-0442-6)
Supplement: Supplementary file 1 — Supplemental description of methods and additional tables and figures. Table S1 Number of systems with discrepancies between demographics characterized using SDWIS’s City served andZip code fields. Table S2 Spearman correlation matrix for county-level predictors, all systems. Table S3 Spearman correlation matrix for county-level predictors, Midwest systems. Table S4 Spearman correlation matrix for county-level predictors, Northeast systems. Table S5 Spearman correlation matrix for county-level predictors, South systems. Table S6 Spearman correlation matrix for county-level predictors, West systems. Table S7 Unadjusted univariate regression model results using nationwide data and county-level demographics. Figure S1 Number of community water systems compiled from SDWIS and subsequently used for regression analyses. Figure S2 Percent of CWSs with mean nitrate ≥5 mg/L by quartile for land use and demographic variables. (PDF 13,659 kb) [file 12940_2018_442_MOESM1_ESM.pdf]

Additional File 1

Environmental justice and drinking water quality: are there socioeconomic disparities in nitrate levels in U.S. drinking water?

Laurel A. Schaider, Lucien Swetschinski, Christopher Campbell, Ruthann A. Rudel

Table of Contents

Methods

Adjustments for wholesale systems pg. 2

Tables

Table S1: Comparison between *City served* and *Zip code* demographics pg. 4

Tables S2-S6: Correlation matrices for regression coefficients (national and each U.S. region) pg. 4

Table S7: Unadjusted regression model results using nationwide data and county-level demographics pg. 7

Figures

Figure S1: Subsetting for community water systems pg. 8

Figure S2: Percent of CWSs with mean nitrate ≥5 mg/L by quartile for land use and demographic variables pg. 9

Adjustments for wholesale systems

In order to characterize the demographics of the entire population served by wholesale community water systems (CWSs), and because purchasing systems were not included in our analysis, it was important that the demographic data for each wholesaler included all of the cities that purchase its water. Nitrate concentrations are not expected to change substantially within distribution systems, so exposure levels should be similar among people served directly by a wholesale system and downstream customers served by water systems that purchase their water from wholesalers. However, SDWIS data for wholesale water systems rarely reflected the cities served by downstream purchasing systems. For instance, the Massachusetts Water Resources Authority (MWRA), the largest CWS in Massachusetts, indirectly serves 48 cities and towns but only reported one city served (Boston) in SDWIS.

Using SDWIS's *Water System Facility* and *Service Area* modules, we linked each wholesaler to purchasing CWSs that serve either a "residential area" or a "municipality" and obtain water from the wholesaler via a "permanent" service connection. We validated these linkages by comparing them to service connection databases (with information about which systems sell water to other systems) provided to us by three states (Iowa, Rhode Island, and South Carolina). The list of service connections that we derived from SDWIS matched well with the data in the detailed state lists, reproducing 448/511 of the documented service connections from those three states. We then used the list of wholesaler-purchaser service connections to augment our records of the cities served by wholesaler water systems by incorporating all of the cities served by affiliated purchasing systems. Using this approach, we improved our database's representation of the cities served by MWRA to 37/48 cities, and ultimately expanded data on the cities served for 1,245 wholesale systems, 174 of which lacked any city served data prior to considering purchasing water systems. Notably, we did not attempt to link wholesaler CWSs with the counties served by purchasing systems since, in some cases, only a small proportion of residents in secondary counties are served by wholesalers.

We also applied the list of wholesaler-purchaser service connections to amend SDWIS estimates of population served, because some states do not include customers of downstream purchasing systems

when reporting a wholesaler's population served. For instance, while SDWIS accurately reported that MWRA served 2.4 million people in its service area, the Metropolitan Water District of Southern California, the nation's largest water system with 19 million customers, was reported to serve zero people. No single feature in the water system data distinguished systems like the Metropolitan Water District that did not include the populations of downstream customers in estimates of population served.

We adjusted the population served from SDWIS for wholesaler CWSs using three metrics to flag systems that appeared to omit downstream customers. First, based on the observation that some wholesalers identify each purchaser as a single, unique service connection, we flagged all wholesalers with a number of service connections within an order of magnitude of the number of associated purchasing systems. Second, we found that some wholesalers counted every water line within their network (including those managed by a purchasing system) among their service connections but did not incorporate the customers served by purchasers' water lines in the population served. Thus, to be conservative, we also flagged systems with 25 times as many service connections as people served. Lastly, because EPA's definition of a public water system specifies that public water systems must serve at least 25 people, we flagged wholesalers with fewer than 25 customers. For all of these flagged systems that were considered to be candidates for population adjustment, we estimated the population served to be whichever was greater of either the original population served reported in SDWIS or the sum of the populations served by all purchasing systems. After this adjustment, a total of 220 systems (6.7% of wholesale systems) changed their system size designation.

Table S1. Number of systems with discrepancies between demographics characterized using SDWIS's *City served* field and demographics characterized using SDWIS's *Zip code* field.

Number of percentage points difference	Number of parameters[a] different					Total
	1	2	3	4	5	
≥10	5,342	3,116	1,533	727	97	10,815 (57.7%)
≥25	3,435	877	326	51	1	4,690 (25.0%)
≥50	2,094	99	13	–	–	2,206 (11.8%)

N = 18,759 CWSs with complete demographic information gathered from the *City served* field and the *Zip code* field corresponding to the mailing address of the administrative personnel.

[a]Parameters evaluated are those collected from the U.S. Census Bureau: Percent Black (non-Hispanic), Percent Hispanic, Percent poverty, Percent home ownership, and Percent urban households

Table S2. Spearman correlation matrix for county-level predictors, all systems. System size included as a numerical category (1=very small, 5 = very large).

	% Black	% Hispanic	% Poverty	% Home owner-ship	% Urban house-holds	% Cropland	Livestock per 100 acres	System size
% Black	1							
% Hispanic	0.131	1						
% Poverty	0.253	0.095	1					
% Home ownership	-0.254	-0.410	-0.369	1				
% Urban households	0.354	0.399	-0.192	-0.409	1			
% Cropland	-0.023	-0.149	-0.027	0.109	-0.140	1		
Livestock /100 acres	-0.093	-0.003	-0.014	0.128	-0.106	0.700	1	
System size	0.096	-0.046	0.078	0.024	-0.005	0.077	0.050	1

Table S3. Spearman correlation matrix for county-level predictors, Midwest[a] systems.

	% Black	% Hispanic	% Poverty	% Home owner-ship	% Urban house-holds	% Cropland	Livestock per 100 acres	System size
% Black	1							
% Hispanic	0.337	1						
% Poverty	0.236	-0.056	1					
% Home ownership	-0.537	-0.294	-0.448	1				
% Urban households	0.635	0.335	-0.070	-0.338	1			
% Cropland	-0.070	0.217	-0.222	-0.026	-0.184	1		
Livestock /100 acres	-0.186	0.127	-0.150	0.030	-0.206	0.578	1	
System size	0.096	-0.046	0.078	0.024	-0.005	0.077	0.050	1

[a]States included in the Midwest are: IL, IN, IA, KS, MI, MN, MO, NE, ND, OH, SD, and WI

Table S4. Spearman correlation matrix for county-level predictors, Northeast[a] systems

	% Black	% Hispanic	% Poverty	% Home owner-ship	% Urban house-holds	% Cropland	Livestock per 100 acres	System size
% Black	1							
% Hispanic	0.745	1						
% Poverty	-0.028	-0.243	1					
% Home ownership	-0.391	-0.310	-0.359	1				
% Urban households	0.606	0.629	-0.389	-0.301	1			
% Cropland	0.141	-0.039	0.256	-0.011	-0.103	1		
Livestock /100 acres	0.014	-0.119	0.165	-0.045	-0.133	0.897	1	
System size	0.123	0.065	0.015	-0.031	0.120	0.003	-0.022	1

[a]States included in the Northeast are: CT, ME, MA, NH, NJ, NY, PA, RI, and VT

Table S5. Spearman correlation matrix for county-level predictors, South[a] systems

	% Black	% Hispanic	% Poverty	% Home owner-ship	% Urban house-holds	% Cropland	Livestock per 100 acres	System size
% Black	1							
% Hispanic	-0.271	1						
% Poverty	0.362	-0.234	1					
% Home ownership	-0.351	-0.243	-0.360	1				
% Urban households	0.081	0.418	-0.378	-0.372	1			
% Cropland	0.001	0.004	0.002	-0.017	-0.218	1		
Livestock /100 acres	-0.379	0.279	-0.090	0.149	-0.083	0.396	1	
System size	-0.008	-0.104	0.140	0.010	-0.078	0.024	0.025	1

[a]States included in the South are: AL, AR, DE, FL, GA, KY, LA, MD, MS, NC, OK, SC, TN, TX, VA, and WV

Table S6. Spearman correlation matrix for county-level predictors, West[a] systems

	% Black	% Hispanic	% Poverty	% Home owner-ship	% Urban house-holds	% Cropland	Livestock per 100 acres	System size
% Black	1							
% Hispanic	0.412	1						
% Poverty	0.016	0.456	1					
% Home ownership	-0.569	-0.423	-0.260	1				
% Urban households	0.631	0.305	-0.081	-0.546	1			
% Cropland	0.018	0.190	0.168	-0.245	0.128	1		
Livestock /100 acres	0.159	0.232	0.125	-0.234	0.161	0.768	1	
System size	0.071	0.086	0.037	-0.039	0.075	-0.011	-0.006	1

[a]States included in the West are: AK, AZ, CA, CO, HI, ID, MT, NV, NM, OR, UT, WA, and WY

Table S7. Unadjusted univariate regression model results using nationwide data and county-level demographics.

Variable	Nitrate concentration		Likelihood of high nitrate[a]	
	Percent change (95% CI)	*p*-value	Percent change (95% CI)	*p*-value
Percent Black, non-Hispanic	−1.1 (−1.4, −0.9)	<0.0001	−3.1 (−4.2, −2)	<0.0001
Percent Hispanic	2.0 (1.8, 2.2)	<0.0001	3.0 (2.6, 3.4)	<0.0001
Percent poverty	−0.6 (−1, −0.3)	0.0003	−0.1 (−1.3, 1.1)	0.82
Percent home ownership	−0.3 (−0.6, −0.1)	0.006	−1.8 (−2.6, −1.1)	<0.0001
Percent urban households	0.1 (0.1, 0.2)	<0.0001	0.4 (0.2, 0.5)	<0.0001
System size: Small[b]	4.1 (0.0, 8.5)	0.052	−27.0 (−35.8, −16.9)	<0.0001
System size: Medium[b]	7.7 (1.8, 14.0)	0.010	−38.6 (−50.9, −23.2)	<0.0001
System size: Large[b]	23.2 (15.7, 31.2)	<0.0001	−34.9 (−49, −17.0)	0.0005
System size: V. Large[b]	22.7 (4.4, 44.1)	0.013	−44.1 (−69.8, 3.6)	0.065
Source water: Groundwater[c]	22.5 (16.5, 28.9)	<0.0001	402 (277, 568)	<0.0001
Percent cropland	0.8 (0.7, 0.9)	<0.0001	3.8 (3.5, 4.1)	<0.0001
Livestock per 100 acres	0.5 (0.4, 0.5)	<0.0001	1.1 (0.9, 1.3)	<0.0001
Region: Northeast[d]	37.3 (−23.7, 147)	0.29	−59.7 (−88.4, 40.1)	0.15
Region: South[d]	3.9 (−37.5, 72.7)	0.88	−69.1 (−89.5, -9.1)	0.033
Region: West[d]	70.5 (0, 191)	0.050	10.8 (−63.3, 234)	0.86

[a]Logistic regression; outcome coded as "1" if system-average concentration ≥5 mg/L and "0" otherwise.
[b]Referent group: very small systems. [c]Referent group: surface water. [d]Referent group: Midwest

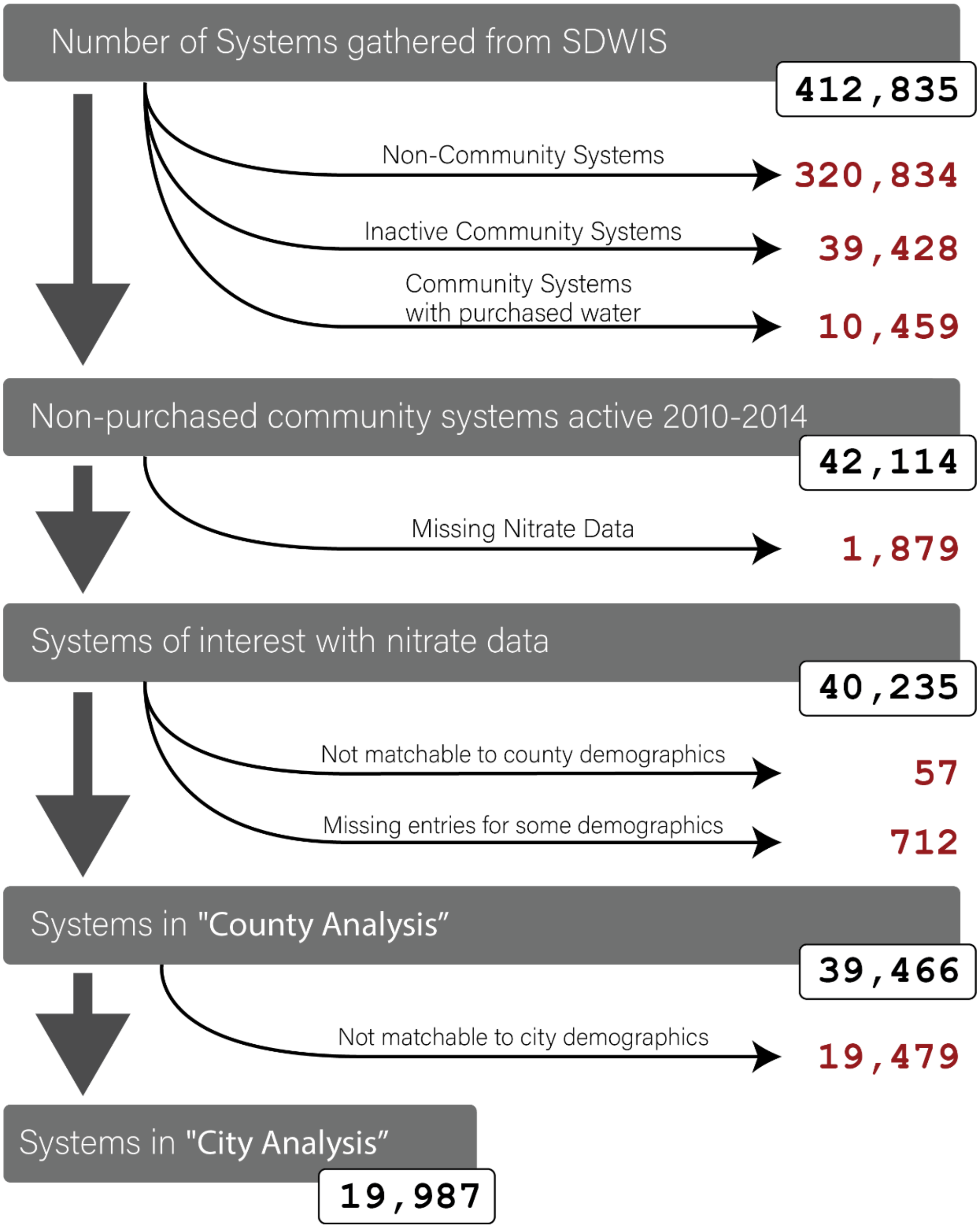

Figure S1. Number of community water systems compiled from SDWIS and subsequently used for regression analyses.

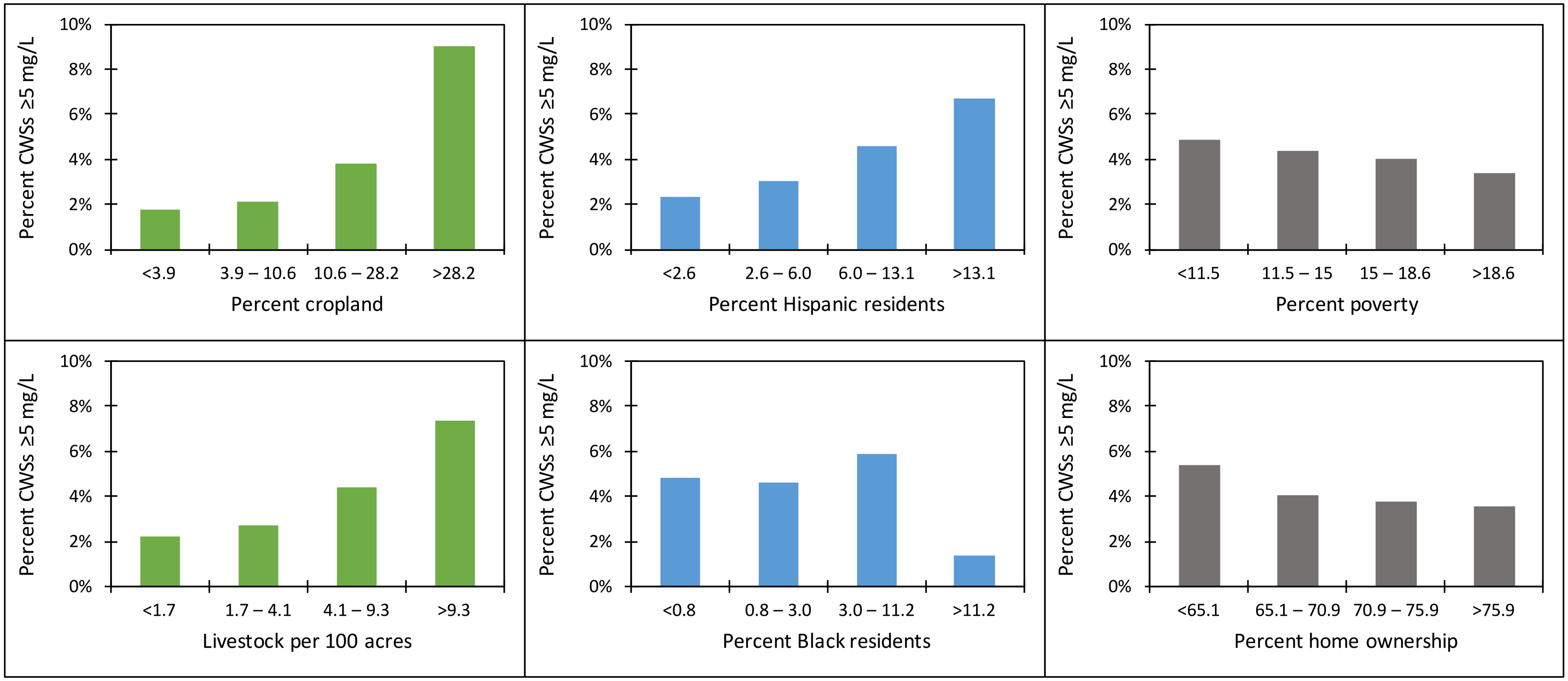

Figure S2. Percent of CWSs with mean nitrate ≥5 mg/L by quartile for land use and demographic variables.
