# Supplementary material for: Environmental justice and drinking water quality: are there socioeconomic disparities in nitrate levels in U.S. drinking water?
Source: Environ Health. 2019 Jan 17;18:3. doi: 10.1186/s12940-018-0442-6 (PMC6335696; doi:10.1186/s12940-018-0442-6)
Supplement: Supplementary file 3 — Data glossary for U.S. community water systems. (PDF 1866 kb) [file 12940_2018_442_MOESM3_ESM.pdf]

Additional file 3 for: Environmental justice and drinking water quality: are there socioeconomic disparities in nitrate levels in U.S. drinking water?

Laurel A. Schaider, Lucien Swetschinski, Christopher Campbell, Ruthann A. Rudel

US community water system (CWS) characteristics

This file represents a portfolio of all Community Water Systems in the United States that were active during the 2010-2014 study period. This includes all presently active systems as well as systems that were deactivated during or after 2010. Systems that purchase their water from another system (those indicated as GWP, GUP, or SWP in the **WS.PRIMARY_SOURCE_CODE** field) are included in this database, but were not analyzed in the study because nitrate testing is not required of systems that purchase their drinking water from another system.

This file includes information provided from the SDWIS API's (basic documentation found here: https://www.epa.gov/enviro/sdwis-model) *Geographic Area* and *Water System* modules, which we aggregated to provide a complete profile of each water system's cities and counties served. This file was supplemented with additional information provided by state drinking water agencies and incorporates updated information regarding the **CITY_SERVED** and **COUNTY_SERVED** variables for Alabama, Kentucky, Pennsylvania, Rhode Island, and Wyoming and updated activity status information for water systems in West Virginia. Amendments were also made to remove multiple instances of a given **CITY_SERVED** or **COUNTY_SERVED** from appearing for a given water system.

Variable definitions:

PWSID - The national identification number for the Public Water System which uniquely identifies the water system within a specific state. Format: SSXXXXXXXXXX where: SS = the Federal Information Processing Standard (FIPS) Pub 5-2 State abbreviation in which the water system is located, or the region number of the EPA region responsible for an Indian reservation, and XXXXXXXXXX = the water system identification code assigned by the State.

PWS_NAME - The name of the water system. The name can be the formal, legal, or common name that is used most generally in referring to the water system. When multiple facilities exist for the water system at different physical locations, the name identifies the means by which the system, as a whole, is generally known.

PRIMACY_AGENCY_CODE - Two character postal code for the state or territory having regulatory oversight for the water system. In this dataset, will only represent a state, but in other datasets can represent the two-digit EPA Region number (if the system is regulated directly by EPA) or NN for Navajo Nation.

PWS_ACTIVITY_CODE - Code that indicates the current activity status of the public water system.

- A - Active
- I/N - Inactive

WS.PWS_DEACTIVATION_DATE - The date in which the water system was reported as being closed/deactivated.

PWS_TYPE_CODE - A system-generated coded value which classifies the water system according to federal requirements. It includes Community Water Systems (CWS), Non-Transient Non-Community Water Systems (NTNCWS), and Transient Non-Community Water Systems (TNCWS).

WS.GW_SW_CODE - Indicates if the water system is considered having ground water (GW) or surface water (GW) source under SDWA.

WS.PRIMARY_SOURCE_CODE - The code showing the differentiation between the sources of water: ground water (GW), groundwater purchased (GWP), surface water (SW), surface water purchased (SWP), groundwater under influence of surface water (GU), or purchased ground water under influence of surface water source (GUP).

WS.POPULATION_SERVED_COUNT - Water system's estimate of the number of people served by the system.

WS.SERVICE_CONNECTIONS_COUNT - Number of service connections to the water system.

AREA_TYPE_CODE - Study created variable categorizing the available documentation for the type area served by a water system.

- CN - System serves a single county
- CN & ZC - System serves a single county and a single zip code
- CT - System serves a single city
- CT & CN - System serves a single city and a single county
- multiCN - System serves multiple counties (and up to one city and/or zip code)
- multiCT - System serves multiple cities (and up to one county and/or zip code)
- multimulti - System serves two or more regions of at least two area types (city, county, zip code)
- multiZC - System serves multiple zip codes (and up to one city and/or county)
- No Geo Available - No information provided on the area served by the water system in either the *Water System* or *Geographic Area* modules

CITY_SERVED - The city served by a water system. Multiple cities are separated by a comma (",").

COUNTY_SERVED - The county served by a water system. Multiple counties are separated by a comma (",").

ZIP_SERVED - The zip code served by a water system. Multiple zip codes are separated by a comma (",").

WS.OWNER_TYPE_CODE - Code that identifies the ownership category of the water system consisting of: F (Federal Government), L (Local Government), M (Public/Private), N (Native American), P (Private), or S (State Government).

WS.IS_GRANT_ELIGIBLE_IND - Code that indicates if the primacy agency has reported the minimum necessary data elements for this water system to include it in grant calculations.

WS.IS_WHOLESALER_IND - Code that indicates whether the system is a wholesaler of water.

WS.IS_SCHOOL_OR_DAYCARE_IND - Code that indicates if the water system's primary service area is a school or daycare as defined by EPA/OGWDW.

SYS_ONLY - Study created variable. Was the system found in the *Water System* module but not the *Geographic Area* module (Y/N)?

GEO_ONLY - Study created variable. Was the system found in the *Geographic Area* module but not the *Water System* module (Y/N)?

CWS facilities & assoc. sellers

This file includes information provided from the SDWIS API's (basic documentation found here: https://www.epa.gov/enviro/sdwis-model) *Water System Facility* module, which details various characteristics of each U.S. water system facility including information on which water systems sell water to facilities. All facilities in this database were active during the 2010-2014 study period. This includes all presently active facilities as well as facilities that were deactivated during or after 2010. This database has been subset to those CWS facilities that purchase water from selling system that is among the 42,114 CWS that were active during our study period (2010-2014) and do not purchase their water (i.e. are not indicated as GWP, GUP, or SWP in the **WS.PRIMARY_SOURCE_CODE** field). In our study, we only associated purchasing system demographics with the wholesaler when the two systems were connected via a permanent service connection (**AVAILABILITY_CODE** = "P") as all other connections were less consistent and it could not be determined if the wholesaler reliably provided water to the purchasing community year-round.

Variable definitions:

PWSID - The national identification number for the Public Water System which uniquely identifies the water system within a specific state. Format: SSXXXXXXXXXX where: SS = the Federal Information Processing Standard (FIPS) Pub 5-2 State abbreviation in which the water system is located, or the region number of the EPA region responsible for an Indian reservation, and XXXXXXXXXX = the water system identification code assigned by the State. In this database, represents the buying facility's PWSID.

PRIMACY_AGENCY_CODE - Two character postal code for the state or territory having regulatory oversight for the water system. In this dataset, will only represent a state, but in other datasets can represent the two-digit EPA Region number (if the system is regulated directly by EPA) or NN for Navajo Nation.

PWS_ACTIVITY_CODE - Code that indicates the current activity status of the public water system.

- A - Active
- I/N - Inactive

PWS_DEACTIVATION_DATE - The date in which the water system was reported as being closed/deactivated.

PWS_TYPE_CODE - A system-generated coded value which classifies the water system according to federal requirements. It includes Community Water Systems (CWS), Non-Transient Non-Community Water Systems (NTNCWS), and Transient Non-Community Water Systems (TNCWS).

FACILITY_ID - Water system facility ID that, when used with the PWSID, uniquely identifies a water system facility.

FACILITY_NAME - Name of water system facility.

FACILITY_TYPE_CODE - Code that Indicates the type of water system facility.

FACILITY_ACTIVITY_CODE - Code that indicates the current activity status of the facility.

- A - Active
- I - Inactive

FACILITY_DEACTIVATION_DATE - The date in which the facility was reported as being closed/deactivated.

AVAILABILITY_CODE - Code for how the source water is utilized by a water system. E (emergency), I (interim), O (other), P (permanent), S (seasonal).

WATER_TYPE_CODE - Indicates the source of the water - GW for Ground Water, SU for surface water, or GU for ground water under the influence of surface water.

IS_SOURCE_IND - Indicated as "Y" is the Water System Facility is designated a source (either a Consecutive Connection (CC), Infiltration Gallery (IG), Intake (IN), Non-piped (NP), Roof Catchment (RC), Reservoir (RS), Spring (SP), Well (WL), or Non-piped non-purchased (NN)).

SELLER_PWSID - PWSID of the water system that is selling water to this system through this interconnection. Is also the "upstream" water system to the parent of this facility.

SELLER_PWS_NAME - PWS Name of the water system that is selling water to this system through this interconnection. Is also the "upstream" water system to the parent of this facility.

SELLER_TREATMENT_CODE - Code that indicates whether the seller is or is not treating the source or whether the seller treatment status is unknown. Applies only to source facilities.

- F - Treated by seller including SWT
- G - Treated by seller with 4-log virus for GWR
- N - No, not treated
- Y - Partially treated by seller

Service areas of purchasing CWSs

This file includes information provided from the SDWIS API's (basic documentation found here: https://www.epa.gov/enviro/sdwis-model) *Service Area* module, which details type of areas served by water systems in the U.S. Included in this dataset are all "primary service areas" (or all service areas in circumstances where no service area was designated as a primary) for the purchasing systems connected to a wholesaler through a permanent service connection. In our analysis, we only associated purchasing system demographics with the wholesaler when the purchasing system served a "Residential Area" or "Municipality" (in the **SERVICE_AREA_DESC** field), as other areas were not thought to be representative of the entirety of the demographics of the purchasing community's cities served.

Variable Definitions:

PWSID - The national identification number for the Public Water System which uniquely identifies the water system within a specific state. Format: SSXXXXXXXXXX where: SS = the Federal Information Processing Standard (FIPS) Pub 5-2 State abbreviation in which the water system is located, or the region number of the EPA region responsible for an Indian reservation, and XXXXXXXXXX = the water system identification code assigned by the State. In this database, represents the buying facility's PWSID.

PRIMACY_AGENCY_CODE - Two character postal code for the state or territory having regulatory oversight for the water system. In this dataset, will only represent a state, but in other datasets can represent the two-digit EPA Region number (if the system is regulated directly by EPA) or NN for Navajo Nation.

PWS_ACTIVITY_CODE - Code that indicates the current activity status of the public water system.

- A - Active
- I - Inactive

PWS_TYPE_CODE - A system-generated coded value which classifies the water system according to federal requirements. It includes Community Water Systems (CWS), Non-Transient Non-Community Water Systems (NTNCWS), and Transient Non-Community Water Systems (TNCWS).

SERVICE_AREA_TYPE_CODE - 2 character abbreviation for service area type.

SERVICE_AREA_DESC - Description of service area.

IS_PRIMARY_SERVICE_AREA_CODE - Indicated "Y" if the service area in question is the primary area type served by the system. Otherwise, all service areas associated with a system are listed.
